# Catalyst- and template-free low-temperature *in situ* growth of *n*-type CdS nanowire on *p*-type CdTe film and *p*-*n* heterojunction properties

**DOI:** 10.1038/srep38858

**Published:** 2016-12-13

**Authors:** Ligang Ma, Wenchao Liu, Hongling Cai, Fengming Zhang, Xiaoshan Wu

**Affiliations:** 1 Collaborative Innovation Center of Advanced Microstructures, National Laboratory of Solid State Microstructures, Jiangsu Key Laboratory for Nano Technology, School of Physics, Nanjing University, Nanjing, 210093, China; 2 School of Electronic Engineering, Nanjing Xiaozhuang University, Nanjing, 211171, China

## Abstract

CdS is an important semiconductor used in optoelectronic devices. Simple techniques for growing CdS nanostructures are thus essential at a low cost. This study presents a novel method for growing single-crystal *n*-type CdS nanowires on *p*-type CdTe films by thermal annealing in an H_2_S/N_2_ mixed gas flow, which does not require the help of a catalyst or template. The formation process and growth mechanism of the nanowires are investigated. Well-dispersed whiskerlike CdS nanostructures are obtained at an appropriate annealing temperature and duration. We suggest that the stress-driving mechanism of nanowire formation may contribute to the growth of CdS nanowires, and that the evaporation of Te through the boundaries of the CdS grain seeds plays an important role in the sustainable growth of nanowire. In addition, CdS/CdTe heterojunction device is fabricated on Mo glass. The *I*-*V* characteristic of the heterojunction in dark shows typical rectifying diode behavior. The turn-on voltage can be regulated by annealing conditions. Meanwhile, the obvious photovoltaic effect is obtained on the *in situ* growth heterojunction prepared at low annealing temperature. Hence, this is a new fabricated method for CdTe-based materials in the field of energy conversion.

CdS is a well-studied semiconductor with versatile properties, such as a direct band gap of 2.4 eV at room temperature, a high refractive index, excellent transport properties and good chemical and thermal stability. It has been used extensively in photovoltaic devices, light-emitting diodes in flat panel displays, transistors, and logic gates^1^,^2^,^3^. Low-dimensional CdS nanostructures, such as nanorods, nanowires, and nanotubes, have attracted increasing attention because of their superior optical, physical, electronic and catalytic properties compared with traditional thin film and bulk materials^4^,^5^,^6^. A wide range of applications for nanodevices and nanosystems based on these CdS nanostructures has recently been explored, including a thin film transistor fabricated on CdS nanoribbons^4^, optical active switching on a single CdS nanowire^7^, hydrogen generators^8^, and CdS nanopillar-array photovoltaics^9^.

Various fabrication methods have been reported for CdS nanostructures, including thermal evaporation^10^, hydrothermal method^11^, two-step metal-organic chemical vapor deposition process^12^, solvothermal synthesis^13^, electrochemical growth method^14^, template method^9^, and chemical vapor deposition (CVD)^15^,^16^,^17^. Although each of these methods has its own advantages, most involve a solution process or organic solvents^13^,^18^, and the reaction process is uncontrollable. The CVD method is known to be effective in the large-scale fabrication of CdS nanostructures. However, due to the high sublimation temperature of CdS powder, the growth temperature of CdS nanostructures is as high as 800 °C^12^,^19^, and the growth process requires the use of an Au catalyst^20^. Unfortunately, Au functions as an impurity, trapping electrons and holes in the nanostructure, and affecting the performance of the device. Templates, such as anodic aluminum oxide^9^,^21^, which are used to prepare nanowires, can also yield better CdS arrays. However, this method also needs Au catalyst to grow nanostructure^9^. So, an effective method of obtaining a high yield of high-quality morphology-tunable CdS nanowires needs to be put forward. Recently, novel method of fabrication CdS nanowire structures has been found, such as catalyst-free template method^22^, *in situ* growth using pulsed laser deposition on low temperature^23^, and assisted synthesis method^24^, and so on.

Herein, we demonstrate for the first time a novel and simple approach to the *in situ* growth of CdS nanowires on a CdTe film surface via annealing in an H_2_S/N_2_ mixed flow at temperatures as low as 450 °C. This low-cost, low-temperature, easy-to-control process requires neither a catalyst and template nor organic solvents and a solution process. On the basis of our experimental results, a possible growth mechanism for CdS nanowires is proposed. More importantly, the substrate we used is a *p*-type CdTe film, and combining with the grown *n*-type CdS nanowires, a promising low dimension CdTe based device is fabricated.

## Results and Discussion

### The Evolution of Morphology

**Figure 1:**
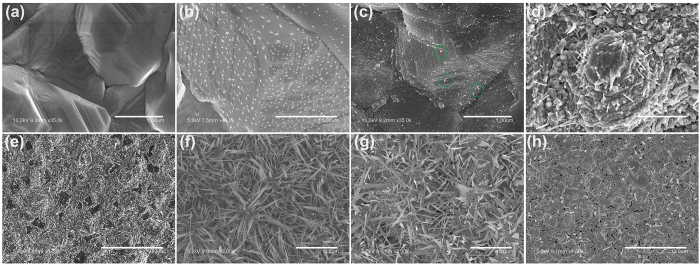
Morphological evolution in the H_2_S gas annealing process. SEM images of CdTe films annealed at (**a**) as-deposited, (**b**) 200 °C, (**c**) 300 °C, (**d**) 400 °C, (**e**) 420 °C, (**f**) 480 °C, (**g**) 530 °C and (**h**) 550 °C.

The morphology of the CdS nanostructures obviously relies on the annealing temperature. A typical morphological evolution of CdS nanowires in the process of annealing are shown in Fig. 1. Initially, the as-deposited CdTe film exhibits a compact and fine-grained morphology with a large grain size ranging from 2 to 4 μm and an obvious grain boundary. After annealing at 200 °C in H_2_S gas flow, the nanoparticles are randomly distributed on the surface of the CdTe film. The X-ray photoelectron spectroscopy (XPS) measurement indicates that nanoparticles are mostly Te particle (see Figure S1 in Supporting Information), and CdS film also is formed at this time (see Figure S2 in Supporting Information). With the annealing temperature increasing, very short nanowires (5~10 nm) unexpectedly appear on the surface of CdTe (300 °C), and the quantity of nanowire is less, which illustrates the initial growth characteristics of the CdS nanostructure, as shown in circle of Fig. 1c. The CdTe grain boundaries become blurred annealing at 400 °C (Fig. 1d).

Then, small grains of CdS grow, aggregate, and form a compact layer on the surface of the CdTe film (annealing temperature at 420 °C). As the annealing temperature further increases to 480 °C, the entire surface is completely covered by the CdS nanowires (Fig. 1f). The nanowires dispersing in length are mostly in the range of 4 and 7 um, and that in diameter is between 50 and 200 nm. The shape of nanowires gradually decreases in diameter from bottom to top, forming a cone. And no any metal nanoparticles are observed at the nanowire tips. When the annealing temperature exceeds 550 °C, the number of whisker-like nanowires decreases sharply, and a large number of voids appears on the surface (Fig. 1h) because Te vapor (the reaction product of CdTe and H_2_S) escapes from the CdTe surface or film during the annealing process.

It is 480 °C that CdS nanowire is formed with a large aspect ratio. And the cross-section image annealed at 480 °C shows three layers: a bottom layer with large grains, a middle CdS layer with relatively small grains, and a top layer with grass-like CdS nanowires (Figure S3 in Supporting Information).

### Microstructure and Crystal Structure

**Figure 2:**
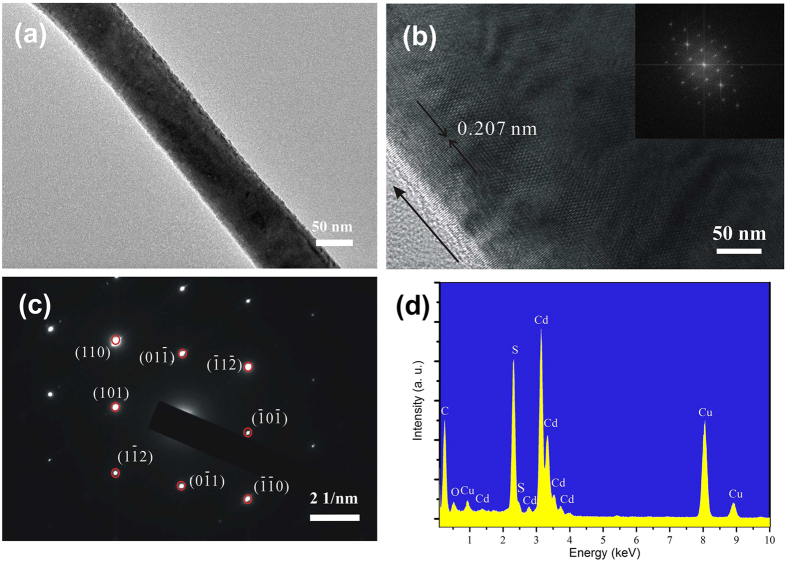
(**a**) Low-magnification TEM image of a single CdS nanowire; (**b**) High-resolution TEM image of the CdS nanowire. Inset is the corresponding Fourier transformation pattern of the entire high-resolution TEM image; (**c**) The SAED pattern recorded by an electron beam perpendicular to the long axis of a single nanowire; (**d**) Energy dispersive spectra taken from the single CdS nanowire.

To explore the microstructures of CdS nanowire, the field emission transmission electron microscopy (FE-TEM) and high-resolution TEM (HRTEM) measurement are conducted. Figure 2a shows a TEM image of a single CdS nanowire. The surface is smooth and the diameter is about 76 nm. The HRTEM image confirms that it is single crystalline, with lattice fringe-resolved *d*-spacing of 0.207 nm, coinciding with the (110) plane spacing in the wurtzite CdS phase (Fig. 2b). The reciprocal lattice peaks are obtained by the fast Fourier transform pattern of the lattice-resolved image (the inset of Fig. 2b), which is in agreement with the corresponding selected area electron diffraction pattern (SAED) pattern shown in Fig. 2c. The SAED is recorded by an electron beam perpendicular to the long axis of a single nanowire and can be indexed along the [−1, 1, 1] zone axis. CdS nanowire is a single crystalline with wurtzite structure and that the (110) plane is the preferred growth direction for the CdS nanowire, as observed in other as-synthesized CdS nanowires^9^,^16^. The energy dispersive X-ray spectrometer (EDS) spectrum taken from the nanowire (Fig. 2d) confirms that it is composed only of Cd and S except those from the Cu grid.

**Figure 3:**
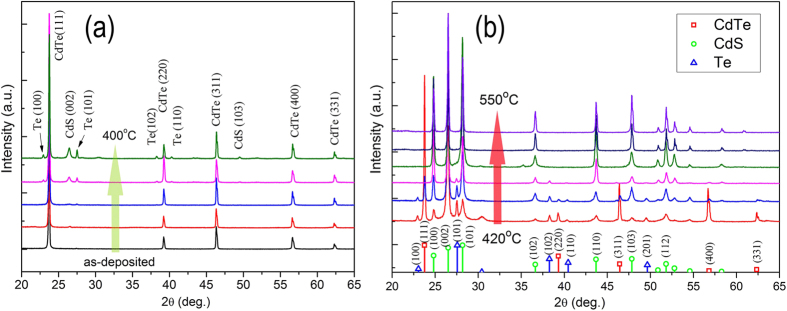
XRD patterns of polycrystalline CdTe film annealed for 1 h at different temperature: (**a**) as-deposited ~ 400 °C, and (b) 420 °C ~ 550 °C. The standard diffraction patterns for the hexagonal wurtzite CdS phase labelled with⃝, the cubic phase CdTe phase labelled with □, and the Te phase labelled with △ are shown at the bottom of the figure.

The influence of annealing temperature on the crystallization behaviors are also characterized by X-ray diffraction (XRD). Figure 3 shows the XRD patterns of CdTe film and CdTe film annealed at different temperature from 200 °C to 550 °C. As for as-deposited CdTe film, the XRD pattern shows that all the diffraction peaks match those of the cubic phase structure of CdTe film (JCPDS card No. 15–0770)^25^.

**Figure 4:**
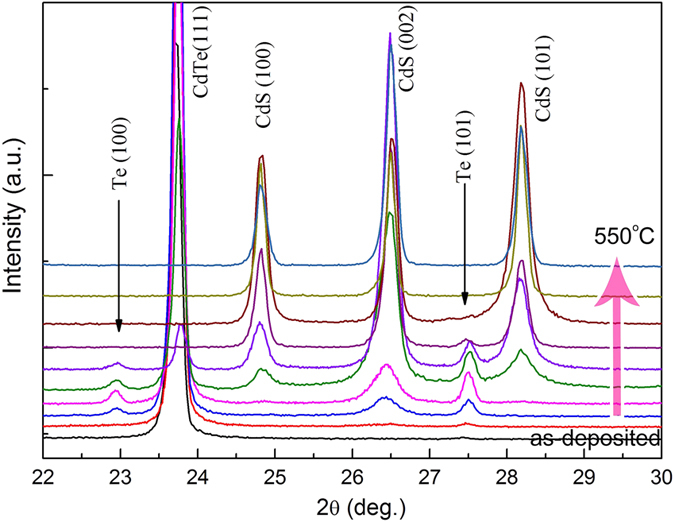
The enlarged XRD patterns between 20~30°.

After annealing in H_2_S gas flow, the XRD patterns contain three phases: the cubic CdTe, the hexagonal CdS, and the Te. And the CdS phase dominates at the expense of CdTe phase, while the CdTe phase gradually disappears with the increase of the annealing temperature. The Te phase increases when annealing temperature is below 420 °C and then become weak and finally disappears. This can be clearly seen in the enlarged XRD patterns between 20–30° (Fig. 4). The diffraction peaks of CdS can be indexed to the wurtzite phase, and without the cubic CdS phase is observed. The sharp and intense peaks suggest that the CdS nanowires are crystalline, which is further confirmed by Raman scattering.

**Figure 5:**
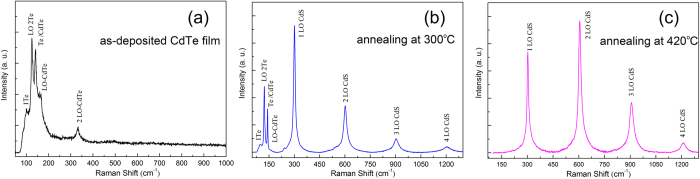
Raman spectra of CdTe films (**a**) as-deposited, (**b**) annealed at 300 °C, and (**c**) annealed at 420 °C.

For as-deposited CdTe film, five Raman peaks (103, 124, 140, 165, and 333 cm^−1^) are observed in Fig. 5a. The Raman peaks located at 165 and 333 cm^−1^ correspond to the LO and 2LO modes of CdTe, respectively. The Raman peaks located at 124 and 103 cm^−1^ correspond to the A_1_ (Te) and LO-TA (CdTe) mode, respectively. And the Raman peak located at 140 cm^−1^ corresponds to the E (Te) or TO (CdTe) mode. These Raman peaks belong to the characteristics peaks of CdTe^26^,^27^. However, Raman scattering undergone a gradually change during annealing process under H_2_S atmosphere. After annealing at 300 °C, the corresponding Raman intensity of CdS is stronger in comparison with abating CdTe Raman peaks, as seen in Fig. 5b. When the annealing temperature increase to 420 °C, more and more CdS are formed during the annealing process. Resonant Raman scattering peaks (302, 605, 907, 1208 cm^−1^) are observed which correspond to the LO, 2LO, 3LO and 4LO modes^28^,^29^ of the CdS film; no signal corresponding to CdTe is detected (Fig. 5c). Linewidth of CdS peak narrow and its line shape is symmetric. This change process is consistent with the results of XRD pattern.

**Figure 6:**
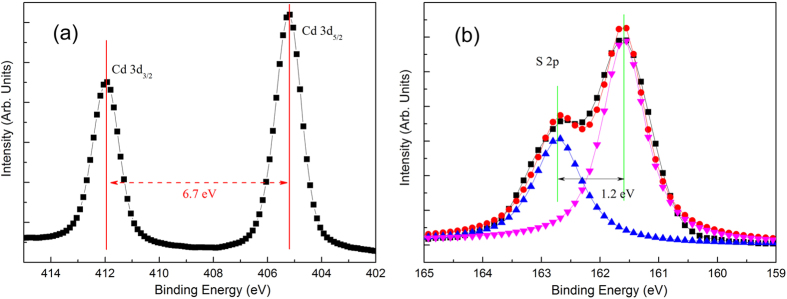
(**a**) XPS of Cd 3d core-level spectra and (**b**) S 2p core-level spectra of a sample annealed at 480 °C.

Survey XPS spectrum for the samples annealed at various temperature were shown in Figure S4 in Supplementary. Fig. 6a and b show XPS of the Cd 3d and S 2p core-level for a sample annealed at 480 °C, which scan over a small energy window at a high resolution, calibrated by the binding energy of C 1 s (285.0 eV). The binding energies of the Cd 3d_5/2_ and the Cd 3d_3/2_ peaks are 405.2 and 411.9 eV, as seen in Fig. 6a, which is consistent with energy splitting of 6.7 eV between the Cd 3d_3/2_ and the Cd 3d_5/2_ states. These binding energy values belong to the CdS compound^30^. A split occurs in the S 2p core-level in Fig. 6b. Gaussian fittings are used to fit the S 2p peaks. The S 2p core-level consists of two peaks at 161.5 eV and 162.7 eV, attributing to S 2p_3/2_ and the S 2p_1/2_ states. The value of the binding energy split is near to 1.2 eV and the area ratio is close to 2:1, which are consistent with S 2p core-level of CdS^31^,^32^. Thus, the formation of CdS is identified not only from the S signal but also from the Cd signal. From the above discussions, we conclude that Cd and S atoms form a CdS over-layer on the surface of the CdTe due to sulphurization.

### Formation Mechanism

Several mechanisms have been proposed to understand the growth of nanostructure, such as the vapor-solid-solid^33^, the vapor-liquid-solid^34^, the solution-liquid-solid^35^, and the oxide-assisted mechanism, etc. However, all of the above mechanisms do not explain the growth of CdS nanowires in our experiment. No metal catalyst is used. No solution phase is appeared in the experiments. No oxygen is involved in the annealing procedure. A tentative theory of the whisker-nanowire growth model proposed by Eshelby^36^ may explain the growth of our nanowires. That is to say, stress may drive whisker growth. The formation of many nanowire materials, including ZnO^37^, Si^38^, Sn nanowire^39^, CuO nanowire^40^,^41^, and α-Fe_2_O_3_^42^,^43^, can be explained by the stress-driven model. Kumar *et al*.^40^ and L. Yuan *et al*.^41^ proposed that the growth of CuO nanowires using thermal oxidation of copper foils in air is attributed to the stress-driven model. The volume change associated with the solid-state transformation at the CuO/Cu_2_O interface produces compressive stresses. A stress-driven grain-boundary diffusion followed by rapid surface diffusion of cations on the sidewall of nanowires is developed to account for CuO nanowire growth. In addition, Fe can be oxidized to form Fe_2_O_3_/Fe_3_O_4_/FeO/Fe layered structure, followed by Fe_2_O_3_ nanowire growth on the outer layer of hematite^43^. The compressive stresses generated by the volume change accompanying the Fe_2_O_3_/Fe_3_O_4_ interface reaction stimulate Fe_2_O_3_ nanowire formation.

In our case, the reaction that forms the CdS nanowires can be described by the following scheme:


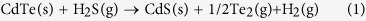


The evaporation temperature of CdS is higher than the annealing temperature in our experiments. However, the evaporation temperature of Te is lower than the annealing temperature. As shown in the Fig. 1h, there are obvious holes on the film surface because Te vapor escapes from the CdTe surface or film during the annealing process. Here, the flow of H_2_S/N_2_ mixed gas is a key factor in the growth process, which can help Te vapor to solidify on the downstream side of the tubular furnace as the low temperature. Thus in position B on the Si substrate the compounds contain Te, S, and O elements except Cd element, which can be found from the result of the EDS spectra (see Supporting Information in Figure S5). All of above analysis indicates that the Te in the CdTe is replaced by S, forming CdS.

**Figure 7:**
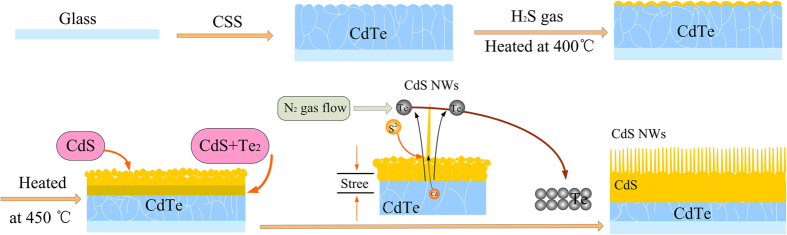
Schematic illustration of growth mechanism and the atom transport mechanisms of CdS nanowire.

The formation of CdS nanowires is shown schematically in Fig. 7. Stress mainly originates from the lattice mismatch between the CdTe film and the CdS film. With the chemical reaction continuing, the stress accumulates to a critical level. Then the strain is released through Te vapor escaping from the surface and the spontaneous growth of nanowires. Small CdS particles function as a nucleation center before growing into CdS nanowires.

Here, the growths of nanowires occur on their tip, and the material transport is from the roots. A reasonably efficient transport channel is required for the feeding stocks, e.g., Cd^2+^, across or through the nanowires. To sustain the nanowires’ growth, S^2−^ is adsorbed on the nanowire tip and Cd^2+^ is transported from the CdTe substrate to the tip with the flow of Te gas. Thus, the shape of the nanowires is controlled by the Cd^2+^ diffusion rate. In the initial stage, the Cd^2+^ diffusion rate is high because of the high evaporation rate of the Te vapor and the short diffusion length. The diameter of the nanowires at the root is thus relatively large. As the CdS nanowires grow longer and the evaporation rate of the Te gas decreases, the Cd^2+^ supply that sustains the uniform growth of the nanowires becomes shorter and shorter, resulting in the formation of a nano-cone structure with a sharp tip. At high temperature, the Te quickly evaporates due to the violent reaction of H_2_S and CdTe. Therefore, the density of the nanowires greatly decreases, which is consistent with the result of our SEM morphologic examination. The proposed mechanism suggests the possible extension of the method to other materials.

### Optical Properties

**Figure 8:**
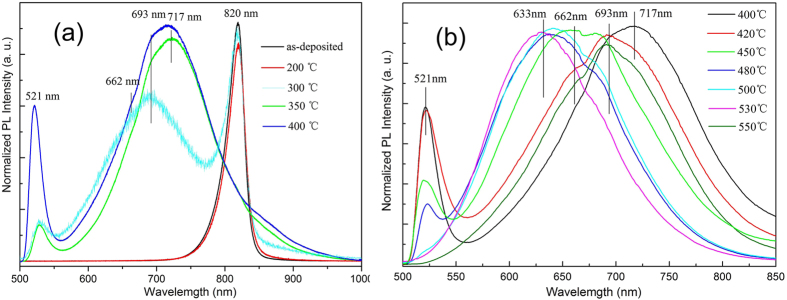
Room-temperature normalized photoluminescence spectra of CdTe films annealed at different temperature.

The annealing temperature dependence of photoluminescence (PL) spectra is shown in Fig. 8. For as-deposited CdTe film and sample annealed at 200 °C, there is an emission peak (820 nm) whose corresponding phonon energy is about 1.51 eV as previous result of the other literature^44^. When the annealing temperature is 300 °C, PL peaks locate at three distinct wavelengths, namely, at ~521 nm, ~693 nm and ~820 nm. This remarkable ternary PL originates from the CdS and CdTe, which implies that the radiative recombination of injected charge carriers can independently take place. In addition, when the annealing temperature at 350 °C, the peak located at 820 nm disappears, and a sharp emission located at 521 nm and a broad peak located at 717 nm are observed. With the annealing temperature further increased to 400 °C, the additional band located at 662 nm appears in the PL spectra. The sharp emission peak at 521 nm (2.38 eV) is assigned to the near band edge emission from the CdS film. And this emission peak becomes weaker and then disappears at annealing temperature higher than 500 °C, which is because that the intrinsic emission peak of CdS (521 nm) is submerged in the strong red emission band^45^. A broad emission peak is centered at 720 nm, and as the annealing temperature increases it shifts gradually to 632 nm, and then to 692 nm. Broad emission is composed of several peaks for CdS films at annealing temperature below 450 °C, i. e., 721 nm (1.72 eV), 688 nm (1.80 eV), and 662 nm (1.87 eV). With the increasing annealing temperature, broad emission peak changes into two overlapping peaks.

Ikhmayies *et al*.^46^ performed PL measurements through the glass on the front surface of the CdS/CdTe solar cell and observed some similar bands in the photoluminescence spectra. They assigned the 1.6~1.8 eV peaks to the emission from the Te complexes in the CdS film, as a result of Te diffusion into the CdS film. We believe that the emission located at 1.72 eV is from the formation of the CdS_1−*x*_Te_*x*_ compound. When the temperature is higher than 420 °C, the surface of the CdTe is replaced by CdS particles or CdS nanowires. The peak located at 662 nm (1.87 eV) is attributable to the CdS nanowire surface states^47^. And the emission peak located at 633 nm is related to morphology.

### Heterojunction Properties

**Figure 9:**
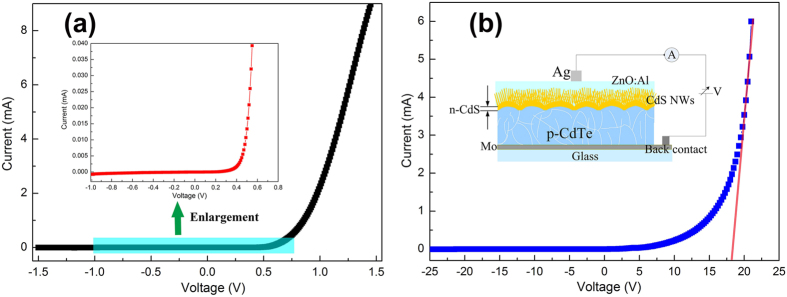
(**a**) *I~V* characteristics under the dark environment of the CdS/CdTe heterojunction fabricated at 370 °C. The inset shows an enlarged curve in the vertical axis direction; (**b**) *I–V* characteristics under the dark environment of the CdS/CdTe heterojunction fabricated at 450 °C. The inset shows a schematic illustration of the electrical measurement system.

In order to further investigate the electrical properties of CdS film/CdS nanowire grown on *p*-CdTe film, a heterojunction device is assembled (inset of Fig. 9b). Figure 9a and b display the current versus voltage (*I–V*) curves under the dark environment of CdS/CdTe heterojunction prepared in two different conditions. The heterojunctions show different properties. As for heterojunction fabricated at 370 °C (Fig. 9a), I–V curve shows a fairly good rectifying behavior. The turn-on voltage is around 0.5 V. The rectification ratio (the forward-to-reverse current ratio) is as high as 3324 at relatively low bias voltages of ±1 V.

However, heterojunction fabricated at 450 °C shows that the turn-on voltage is around 17~20 V and the leakage current for the heterojunction is very weak when the applied reversed bias voltage is −25 V (Fig. 9b). And the rectification ratio is also calculated at relatively low bias voltages of ±20 V that the value is 1740. It is clearly seen from the SEM patterning that at low temperature (370 °C) CdS film is formed on CdTe surface, while at high temperature (450 °C) CdS nanowire appeares. This phenomenon indicates the large turn-on voltage is due to the existence of nano *p*-*n* junction. In view of *p*-type semiconductor for CdTe film, the good rectifying behavior in the fabricated heterostructure indicates that resultant CdS film/CdS nanowire is *n*-type semiconductor. Furthermore, a good heterojunction interface between CdS and CdTe is perfectly formed which also offers a practical application of CdTe-based devices.

**Figure 10:**
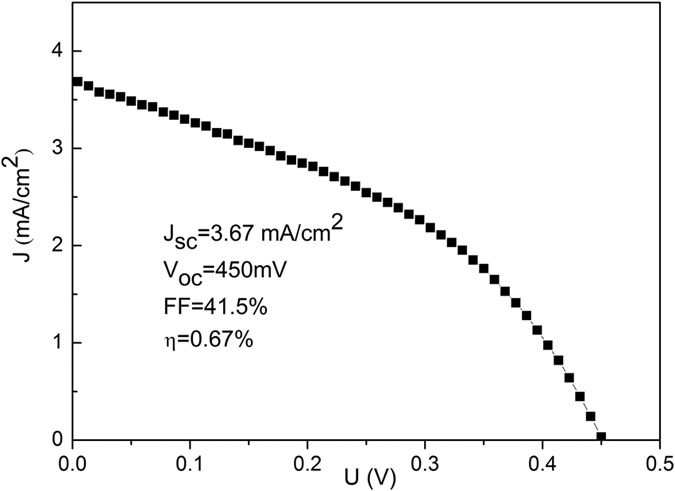
*J–V* curve measured under simulated AM 1.5 illumination for CdTe/CdS solar cell based on the CdTe thin film annealed at 370 °C in the H_2_S gas. The structure of solar cell is Mo/CdTe/CdS/ITO. The inset shows the detailed performance parameters.

We measured the current density-voltage (*J–V*) curves under simulated AM 1.5 (100 mW/cm^2^) illumination for CdTe/CdS heterojunction prepared in two different conditions (annealed at 370 and 450 °C). As for heterojunction prepared at 370 °C, the obvious photovoltaic effect is observed, as shown in Fig. 10. The device shows the short circuit current density (J_sc_), the the open-circuit voltage (V_oc_), the fill factor (FF), and the conversion efficiency (η) to be 3.67 mA/cm^2^, 450 mV, 41.5%, and 0.67%, respectively. But, heterojunction prepared at 450 °C doesn’t show the photovoltaic effect (data not shown here). This is because the thickness of CdS layer in higher annealing temperature is too thick, which block the light into the heterojunction. This method provides a novel fabricated approach for CdTe-based materials in the field of energy conversion.

## Conclusion

We introduce a novel, simple, and low-temperature method for the synthesis of large-scale and high-density single-crystal hexagonal wurtzite structure CdS nanowires based on CdTe film. The merit of this method is that the process requires neither a catalyst and template nor organic solvents and a solution process. The replacement reaction occurs on the CdTe surface to form a CdS layer and CdS nanowire. The surface morphology of the CdS is strongly affected by the annealing temperature and the annealing time. The strain of the CdS on the CdTe may be the driving force in the formation of nanowires. The photoluminescence spectra measured at room temperature reveals green and red emission bands. The *p*-CdTe/*n*-CdS heterojunction exhibits a distinctly rectifying behavior and low saturation current. And the turn-on voltage can be regulated by annealing conditions. This research may be useful for solar energy applications of CdTe.

## Methods

### Synthesis of CdTe film

In a typical synthesis process, the CdTe films were firstly deposited on soda lime glass substrates (or silicon substrate, or flexible metal foil substrate) using the home-built closed-space sublimation system in our laboratory. CdTe powders (99.999%, Alfa Aesar) were used as its growth source. The substrates were ultrasonically cleaned in acetone and rinsed in deionized water, and then placed in the deposition chamber. The deposition chamber was initially evacuated and washed several times as the residual pressure was reduced to 1 Pa. High-purity Ar gas was then introduced into the chamber to maintain a reactant pressure of 2 kPa. The source and substrate temperatures were kept at 630 °C and 560 °C, respectively. The CdTe powders were placed in a graphite container, and the distance between the source and the substrate was maintained at 2 mm during deposition. The thickness and roughness of CdTe film were 6.9 μm and 0.4 μm, respectively.

### Synthesis of CdS nanowire

The CdS nanowires were grown in a horizontal quartz tube furnace (OTF 1200X-S, MTI) via annealing under a mixture of H_2_S (5%) and N_2_ (95%). The prepared CdTe film was loaded in the center of a quartz tube (position A in Fiugre S6 in the Supporting Information) and the reaction by-product was collected in location B (downstream part of the quartz tube). Before annealing, the system was flushed several times with an H_2_S/N_2_ mixed gas flow to minimize the oxygen content. In addition, 10 mol/L NaOH solutions were used to absorb the H_2_S off-gas and change it into harmless materials. The CdTe film was then annealed under an H_2_S/N_2_ mixed atmosphere at a flow rate of 12 sccm. The rate of increasing temperature was maintained at 10 °C/min, and the annealing temperature ranged from 200 °C to 550 °C for 1 h. Finally, the sample was cooled to room temperature. A typical optical image of the CdS nanowire on CdTe film is shown in Figure S7 (Supporting Information).

### Characterization methods

The crystal structures were characterized using a Rigaku Dmax-rB X-ray powder diffractometer with Cu Kα radiation (λ = 1.5418 Å). The operating voltage and current were maintained at 40 kV and 60 mA, respectively. FE-SEM was carried out using S-4800 equipment to observe the surface morphology. X-ray photoelectron spectroscopic measurements were performed in a PHI 5000 VersaProbe using a monochromatized Al Kα source to identify the chemical composition of the film and the corresponding electronic valence. The microstructure of a single nanowire was analyzed using FE-TEM, JEOL JEM 2100 at an accelerating voltage of 200 kV, equipped with an EDS to identify the chemical species of the nanowire. For TEM observation, the CdS nanowires on the substrate were ultrasonically dispersed in ethanol for 10 s. An ethanol suspension containing the CdS nanowires was then dropped onto a Cu grid coated with a holey carbon film. PL spectra and Raman measurements were made with a Horiba Jobin Yvon HR800 microscope system. The spectra were excited using the 488 nm line of an air-cooled Ar-ion laser and a charge-coupled device detector. A microscope with a ×100 objective was used to focus the incident laser beam to a spot, and the spot size was estimated to be less than 2 μm. All of the measurements were performed at room temperature.

### CdS/CdTe heterojunction device fabrication

The CdTe film was fabricated on Mo-coated glass substrate, which was used as the back contact electrodes. Then, the annealing treatment in H_2_S atmosphere was performed at 370 °C and 450 °C to form CdTe/CdS heterojunction. The as-grown heterojunction soaked in a saturated CdCl_2_ ethanol solution for 10 min, followed by a annealing for 20 min at 400 °C under the protection of nitrogen. Finally, conductive film, ZnO:Al thin film was deposited on CdS nanowire using atomic layer deposition equipment as the top contact electrode, which formed a ohmic contact between CdS and ZnO:Al. The room temperature dark *I-V* characteristics of device were recorded with a Keithley 2400 Source Meter. The *J*–*V* curves were carried on a Keithley 2400 source measurement unit under AM 1.5 illumination (standard 100 mW/cm^2^) cast by an Oriel 92251A-1000 sunlight simulator calibrated by the standard reference of a Newport silicon solar cell.

## Additional Information

**How to cite this article**: Ma, L. *et al*. Catalyst- and template-free low-temperature *in situ* growth of *n*-type CdS nanowire on *p*-type CdTe film and *p-n* heterojunction properties. *Sci. Rep.* **6**, 38858; doi: 10.1038/srep38858 (2016).

**Publisher's note:** Springer Nature remains neutral with regard to jurisdictional claims in published maps and institutional affiliations.

## Supplementary Material

Supplementary Information
