# Supplementary material for: Catalyst- and template-free low-temperature in situ growth of n-type CdS nanowire on p-type CdTe film and p-n heterojunction properties
Source: Sci Rep. 2016 Dec 13;6:38858. doi: 10.1038/srep38858 (PMC5221564; doi:10.1038/srep38858)
Supplement: Supplementary Information [file srep38858-s1.pdf]

Supporting Information for

Catalyst- and template-free low-temperature *in situ* growth of n-type CdS nanowire on p-type CdTe film and p-n heterounction properties

Ligang Ma[a, b], *Wenchao Liu*[b], *H. L. Cai*[a], *F. M. Zhang*[a], *X. S. Wu*[a*]

[a] Collaborative Innovation Center of Advanced Microstructures, National Laboratory of Solid State Microstructures, School of Physics, Nanjing University, Nanjing 210093, China

[b] School of Electronic Engineering, Nanjing Xiaozhuang University, Nanjing 211171, China

Author to be corresponded: xswu@nju.edu.cn

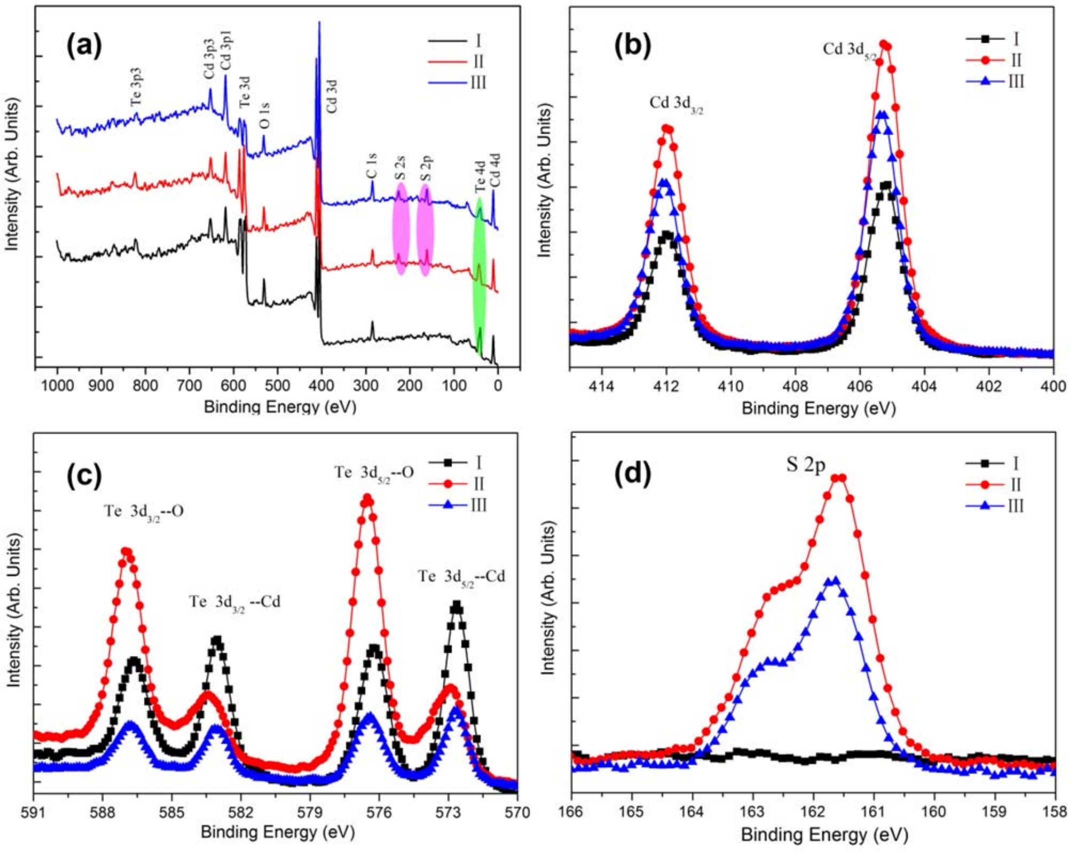

Figure S1 XPS spectrum of (a) survey spectrum, (b) Cd core-level, (c) Te core-level, and (d) S core-level spectra in three kinds of preparation conditions, respectively. CdTe film without any treatment is labeled as Ⅰ, the CdTe film annealed under H_2S

gas at 200 °C is labeled as Ⅱ, and treated by N_2 gas flow followed by the former treatment is labeled as Ⅲ. The detected elements are labeled as shown in Figure.

To confirm element and content of the nanoparticles in Figure 1b, sample annealed in H_2S gas flow at 200 °C are further annealed in N_2 gas flow at 400 °C. The XPS of three kinds of preparation conditions are shown in Figure S4. From the figure, the content of Te element decrease sharply after annealing in N_2 gas flow, while the content of Cd and S element have a little decrease. This is because nano-Te have a low evaporation temperature. It is taken away by air in annealing process in N_2. Therefore, the nanopartical in Figure 1b is mostly Te nanoparticales.

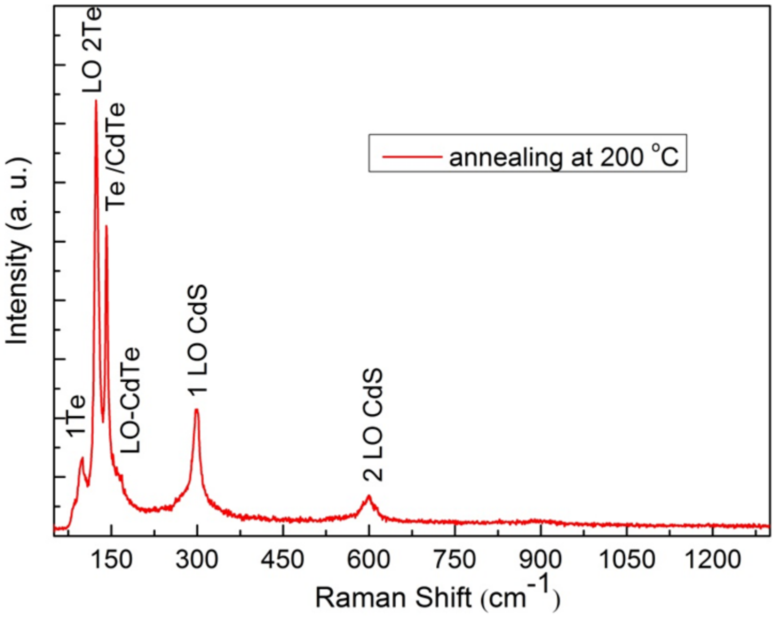

Figure S2. Raman spectra of CdTe films annealed at 200 °C.

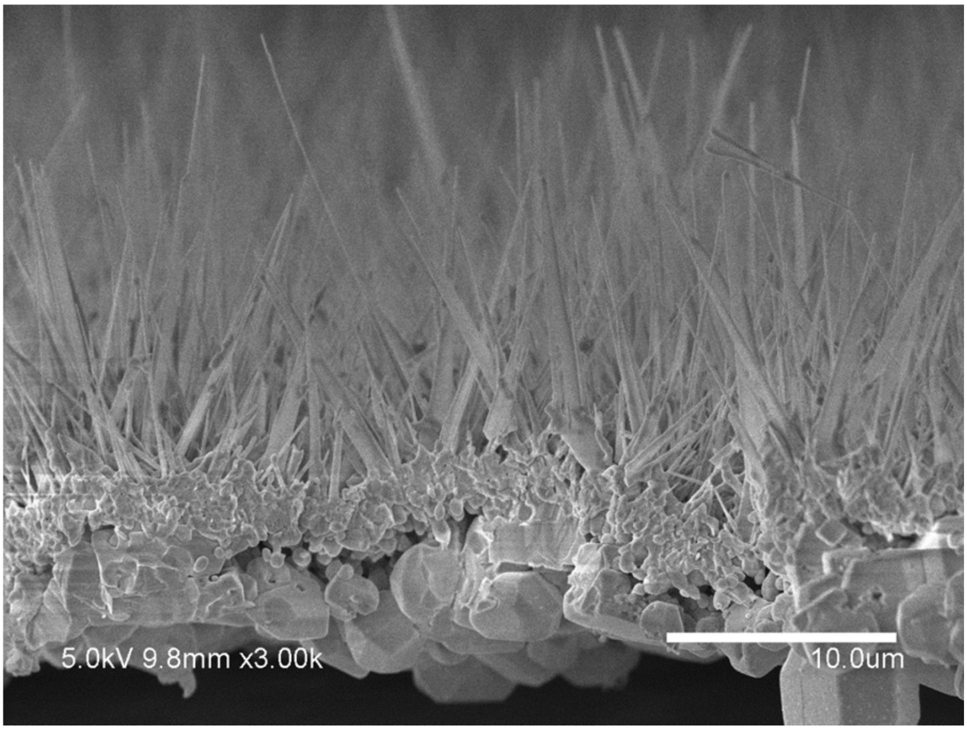

Figure S3 Cross-sectional SEM image showing the CdS and CdTe layers (grown at 480 °C).

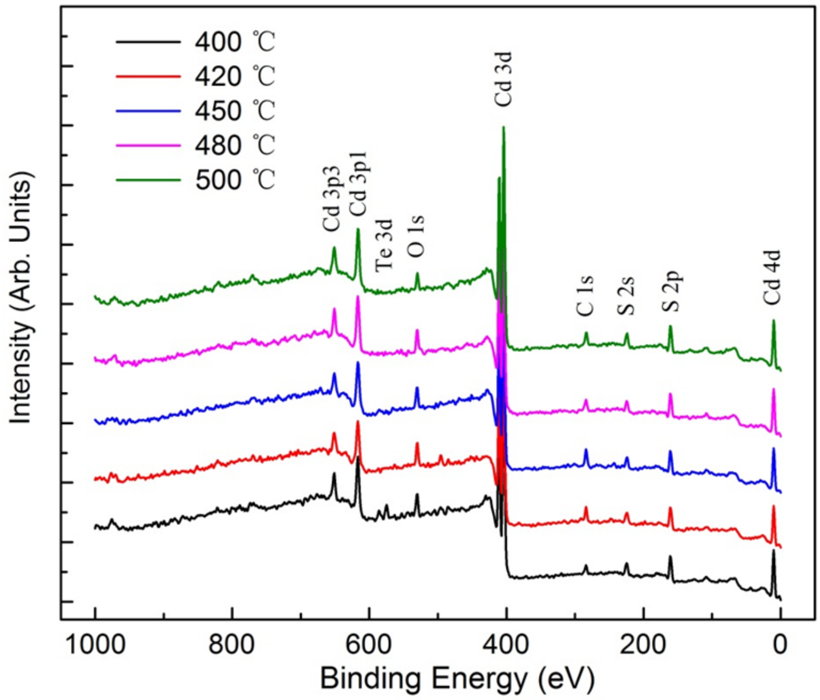

Figure S4. Survey XPS spectrum for the samples annealed at various temperature. The identified peaks are labeled to the various corresponding elements.

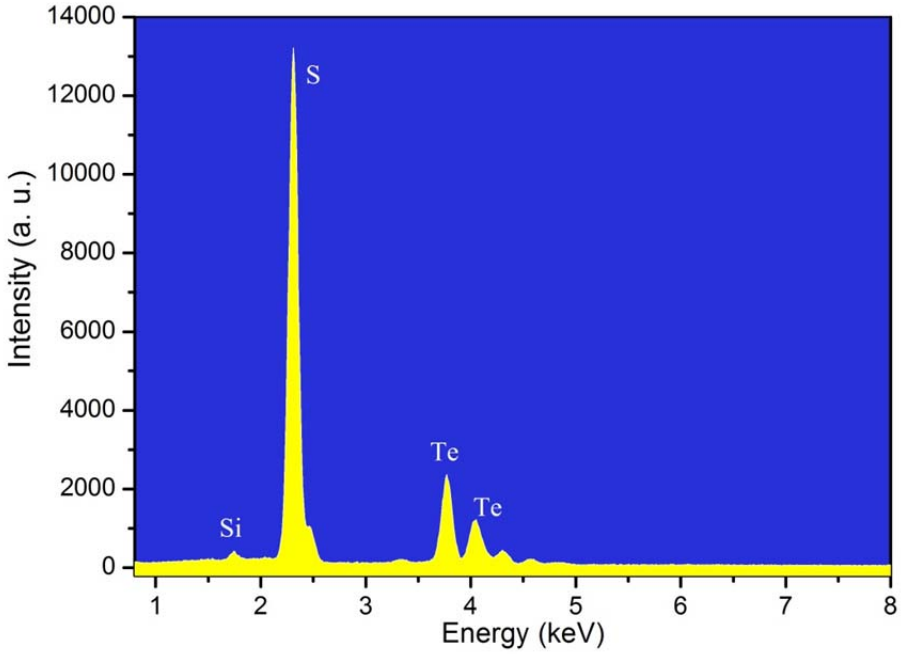

Figure S5. EDS spectra of the products showing positions B seen from Figure S6 (the quartz tube wall downstream).

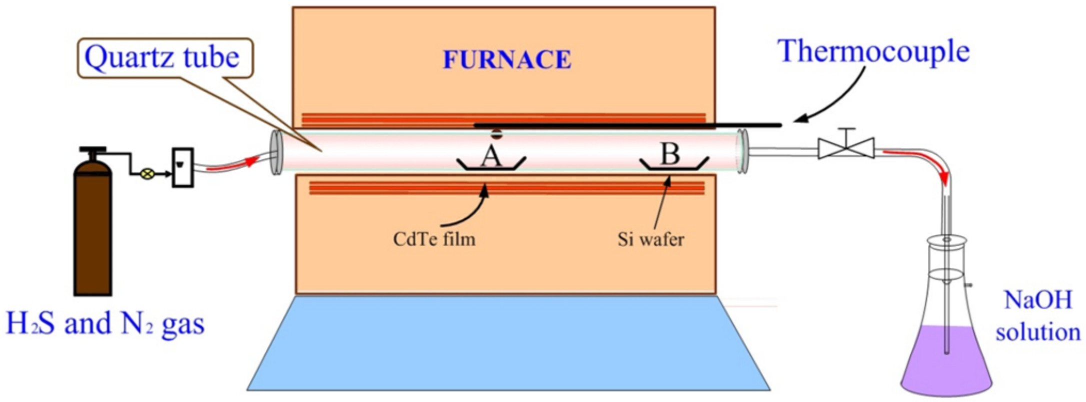

Figure S6. Schematic of the instruments for growing of CdS nanowire.

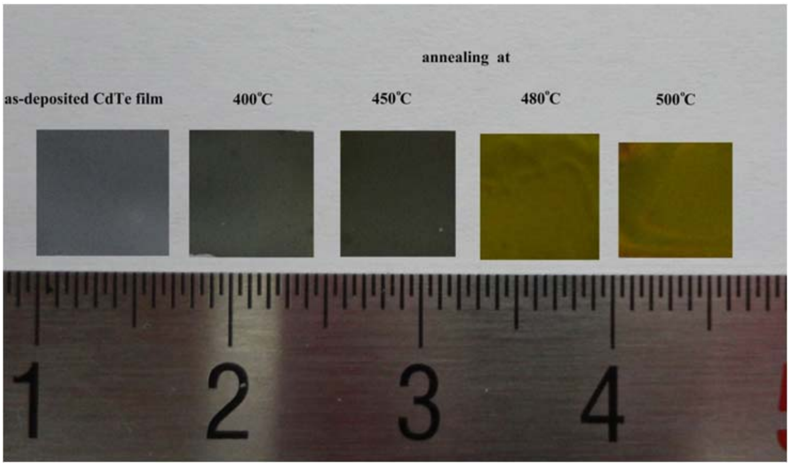

Figure S7 A digital photo shows the color of products changing from black to yellow with increasing the annealing temperature.
